# Imputation of non-genotyped individuals based on genotyped relatives: assessing the imputation accuracy of a real case scenario in dairy cattle

**DOI:** 10.1186/1297-9686-46-6

**Published:** 2014-02-03

**Authors:** Aniek C Bouwman, John M Hickey, Mario PL Calus, Roel F Veerkamp

**Affiliations:** 1 Animal Breeding and Genomics Centre, Wageningen UR Livestock Research, P.O. Box 135, Wageningen 6700, AC, Netherlands; 2 The Roslin Institute, Royal Dick School of Veterinary Studies, The University of Edinburgh, Easter Bush, Midlothian, Scotland, UK

## Abstract

**Background:**

Imputation of genotypes for ungenotyped individuals could enable the use of valuable phenotypes created before the genomic era in analyses that require genotypes. The objective of this study was to investigate the accuracy of imputation of non-genotyped individuals using genotype information from relatives.

**Methods:**

Genotypes were simulated for all individuals in the pedigree of a real (historical) dataset of phenotyped dairy cows and with part of the pedigree genotyped. The software AlphaImpute was used for imputation in its standard settings but also without phasing, i.e. using basic inheritance rules and segregation analysis only. Different scenarios were evaluated i.e.: (1) the real data scenario, (2) addition of genotypes of sires and maternal grandsires of the ungenotyped individuals, and (3) addition of one, two, or four genotyped offspring of the ungenotyped individuals to the reference population.

**Results:**

The imputation accuracy using AlphaImpute in its standard settings was lower than without phasing. Including genotypes of sires and maternal grandsires in the reference population improved imputation accuracy, i.e. the correlation of the true genotypes with the imputed genotype dosages, corrected for mean gene content, across all animals increased from 0.47 (real situation) to 0.60. Including one, two and four genotyped offspring increased the accuracy of imputation across all animals from 0.57 (no offspring) to 0.73, 0.82, and 0.92, respectively.

**Conclusions:**

At present, the use of basic inheritance rules and segregation analysis appears to be the best imputation method for ungenotyped individuals. Comparison of our empirical animal-specific imputation accuracies to predictions based on selection index theory suggested that not correcting for mean gene content considerably overestimates the true accuracy. Imputation of ungenotyped individuals can help to include valuable phenotypes for genome-wide association studies or for genomic prediction, especially when the ungenotyped individuals have genotyped offspring.

## Background

With the reduction in genotyping costs, data on phenotypes are becoming a limiting factor in livestock genetics, especially for traits that are difficult, expensive or invasive to measure (e.g., feed intake). Historical datasets, for instance those used for estimating heritability, often lack genotyping data and the individuals might no longer be available for DNA collection. Imputing genotypes for these phenotyped individuals increases the potential usefulness of these phenotypes, for instance for genome-wide association studies (GWAS) [1-3] or for genomic prediction [4-6]. If a relevant genotyping strategy can be chosen such that imputation accuracy is sufficiently high, imputation of ungenotyped animals might also be of interest for breeding programs to reduce genotyping costs.

The difficulty for imputation lies in the fact that these phenotyped individuals have no genotypes, thus information for imputation has to come from relatives. Often the sires and grandsires of these ungenotyped individuals are genotyped, but also offspring and other relatives might be genotyped or available for genotyping, which enables imputation of ungenotyped individuals.

Several software programs for imputation are available; some programs were designed for human populations and others for livestock populations. Comparisons of imputation programs have been mostly carried out for situations in which low-density genotyped individuals are imputed to high-density genotypes e.g. [7-10]. The performance of different imputation programs depends mostly on the data structure, e.g., density of single nucleotide polymorphism (SNP) panels, size of the reference population, and whether related or unrelated individuals were genotyped. Thus, choosing the best imputation method for a given data set is not straightforward. Population-based imputation programs rely on linkage disequilibrium (LD) information and in general perform well to impute both individuals that are unrelated to genotyped individuals and related individuals, e.g. [8,11-13]. Pedigree-based imputation methods incorporate information from both LD and pedigree relationships for imputation. For imputation of very low-density genotyped animals, e.g., using 384 SNPs, pedigree-based imputation programs appear to be more accurate, especially when more and closer relatives are genotyped [4,14,15]. Only a few pedigree-based imputation programs can impute non-genotyped individuals in the pedigree, e.g., AlphaImpute [4], FImpute [16], FindHap [17], and PedImpute [18]. The accuracy of imputing ungenotyped individuals has not been extensively studied but depends strongly on the number of close relatives that are genotyped [4,10,14,15].

The objective of this study was to investigate the accuracy of imputation of non-genotyped individuals using genotype information from relatives. This paper is based on a real (historical) dataset that includes dairy cows that were phenotyped for feed intake and with part of the dataset genotyped. To evaluate imputation accuracy, genotypes were simulated for all individuals in the pedigree. Different scenarios were evaluated (the actual data scenario, addition of genotypes of sires and maternal grandsires, and addition of offspring genotypes) to assess whether using additional genotype information increases imputation accuracy.

## Methods

### Data

This study was based on a real dataset of dairy cows that were phenotyped for feed intake on three experimental herds in the Netherlands. The dataset consisted of 2365 phenotyped cows with a pedigree of 14 733 individuals. In total, 4097 individuals in the pedigree were genotyped with a 50 k SNP panel, of which 1021 had both phenotypes and genotypes and 3076 had only genotypes. There were 1344 phenotyped cows without genotypes that needed to be imputed, of which 998 had no recent ancestors among the genotyped individuals and 346 had a sire, a dam, grand sires or a combination of these that were genotyped. In addition, 539 of the 1344 non-genotyped cows had at least one genotyped offspring. A more detailed description of the relationships between the genotyped reference population and the 1344 phenotyped cows without genotypes is in Table 1.

**Table 1:** Description of testing and reference sets for each scenario and different testing sets

	**Real**	**SireMGS**^**1**^	**Off0**	**Off1**	**Off2**	**Off4**
Testing set	1,344	1,344	805^2^	805	805	805
Both parents	14	43	25	25	25	25
SireMGS	62	1,258	756	756	756	756
DamPGS	6	0	0	0	0	0
Sire	241	24	15	15	15	15
Dam	23	0	0	0	0	0
Other	998	19	9	9	9	9
Reference set	4,079	4,716	4,716	5,521	6,326	7,936
At least one offspring in reference^2^	539	539	0	805	805	805

^1^Genotypes of sires and maternal grandsires of phenotyped individuals not already included in the real scenario were added; ^2^within the testing set of 1344 cows, 539 had at least one offspring genotyped and were removed from the testing set for the offspring scenarios, hence there were only 805 cows in the testing set in Off0 to Off4.

### Scenarios

To assess the accuracy of imputation based on different genotyped relatives in the real dataset, six scenarios were tested. Our first goal was to determine the accuracy of imputation in the real situation (scenario Real), when the 1344 ungenotyped individuals with phenotypes were imputed using simulated genotypes of all 4097 individuals that were genotyped in the real data. The second goal was to assess whether imputation accuracy increased when genotypes for sires and maternal grandsires of the phenotyped individuals were available (scenario SireMGS). For this purpose, simulated genotypes of sires and maternal grandsires of the ungenotyped individuals were added to the reference population for imputation. This scenario is realistic because in practice, most sires and maternal grandsires are in fact genotyped. The third goal was to assess the increase in accuracy when the ungenotyped individuals had no, one, two or four genotyped offspring in addition to genotyped sires and maternal grandsires. For this scenario, the 805 phenotyped cows without genotypes that had no offspring in the real pedigree were selected as the testing set (no offspring scenario: Off0). For each of these 805 cows, four half-sib offspring were simulated by mating them at random with 60 simulated founder sires. In scenarios four, five and six, simulated genotypes of one (scenario Off1), two (Off2), and four offspring (Off4), respectively, were available for imputation. The genotypes of the sires of the offspring (i.e., the mates of the individuals to impute) were not included in the reference population. Table 1 gives an overview of all six scenarios.

The 1344 individuals phenotyped but not genotyped in the real situation were used as the testing set for the scenarios Real and SireMGS. For scenarios Off0 to Off4, a subset of 805 of these 1344 individuals which did not have offspring in the real pedigree was selected for testing.

### Simulation of genotypes

Genotypes were simulated for all individuals in the pedigree following Daetwyler et al. [19], in ten replicates. Sequence data was simulated for 4000 haplotypes for one chromosome using a coalescent approach [20]. The chromosome was 100 cM long and contained 1.0 × 10^8^ base pairs that were simulated using a per site mutation rate of 2.5 × 10^-8^ and a varying effective population size over time that reflected estimates for a Holstein cattle population [21]: 100 in the final generation of haplotype sequence simulation, 1256 at 1000 years ago, 4350 at 10 000 years ago, and 43 500 at 100 000 years ago.

Simulated base generation haplotypes were then dropped through the pedigree using AlphaDrop [22]. The pedigree comprised the real pedigree structure, with the addition of the four simulated half-sib offspring from 805 phenotyped cows that did not have genotypes nor offspring in the original dataset, and 60 simulated (founder) sires of those offspring. Thus, the final pedigree included 18 053 individuals.

Two thousand bi-allelic SNPs were randomly sampled from the sequence data to represent SNPs from a SNP-chip panel and were used for imputation. The mean minor allele frequency (MAF) across all replicates was equal to 0.23. The MAF of the simulated SNPs for the full pedigree showed a slightly U-shaped distribution, which is expected for a random sample of sequence data, but the distribution was quite similar to the uniform distribution that is commonly observed in real 50 k SNP-chip cattle data e.g. [23]. The LD decay pattern of the simulated SNPs resembled the LD decay in dairy cattle populations.

### Imputation

AlphaImpute, a pedigree-based imputation approach that combines long-range phasing, haplotype library imputation, simple rules and genotype probabilities, was used to impute missing genotypes [4]. AlphaImpute has multiple settings and the defaults described in the manual were used for most parameters, but longer cores were used to reduce the computation time for phasing. The conservative haplotype library imputation step was used to limit the number of haplotypes that get filled in for ungenotyped individuals without using pedigree information. This reduced imputation errors resulting from random matches of incorrect haplotypes with partially imputed genotypes of ungenotyped individuals. Hereafter, we refer to this imputation approach as AlphaImpute phased.

As in Pimentel et al. [5], we found that imputation with simple segregation rules performed better than imputation methods that used LD information. Therefore, AlphaImpute was also used in a setting without phasing, in which case, the program imputed genotypes using basic inheritance rules and segregation analysis, as described by Kerr and Kinghorn [24]. Only family information and allele frequencies were used when running AlphaImpute without phasing, thus no LD, linkage, or haplotype information was used. Hereafter we refer to this imputation approach as segregation analysis.

### Assessing imputation accuracy

Individuals in the testing set were divided into categories according to their relationship with their most recent genotyped ancestor(s) i.e.: both parents genotyped (BothParents), sire and maternal grandsire genotyped (SireMGS), dam and paternal grandsire genotyped (DamPGS), sire only genotyped (Sire), dam only genotyped (Dam), and other (Other). For each scenario and each category, the mean animal-specific imputation accuracy and its standard deviation, the percentage of correctly and incorrectly imputed SNP genotypes per individual and the percentage of genotypes not imputed per individual were calculated across the 10 replicates. The percentage of SNP genotypes not imputed represented genotypes that were set to missing by AlphaImpute because they could not be imputed with sufficient certainty due to insufficient information, e.g. around recombination events or due to uncertainty on which haplotype was inherited from one or both parents. However, these missing SNP genotypes did receive genotype dosage probabilities and these were included when calculating the imputation accuracy. In fact, for all SNPs the genotype dosage probabilities (ranging from 0 to 2) were used, rather than the most likely genotypes.

The animal-specific imputation accuracy was assessed by computing for each individual the correlation of the true genotypes (0, 1, or 2) minus the mean gene content per SNP with the imputed genotype dosages minus the mean gene content per SNP as proposed by Mulder et al. [25]. The correction for mean gene content of each SNP was introduced because different SNPs have different MAF and thus distributions with different means, while the Pearson correlation coefficient assumes that the two variables that are correlated are bivariate normally distributed. Mean gene content was calculated per SNP as the mean of the genotypes represented as 0, 1, and 2 (i.e., 2p, with p representing the frequency of the allele for which the homozygote is coded as 2), and was based on genotyped reference individuals in each scenario. For comparisons with previous studies, we also computed the commonly used uncorrected accuracy of imputation per individual (r_uncorrected_) based on the correlation of true genotypes with imputed genotype dosages. However, it should be noted that this accuracy is biased upward due to differences in MAF between SNPs, especially when imputation accuracy is low, and is therefore less suitable to quantify animal-specific imputation accuracy [25].

Besides computing imputation accuracy and percentages of (in)correct SNP genotypes and genotypes not imputed for each individual, we also computed those parameters for each SNP across individuals. SNP-specific imputation accuracy was defined as the correlation of true genotypes with imputed genotype dosages per SNP across individuals. Also for the SNP-specific imputation accuracy, genotype dosage probabilities were used, rather than the most likely genotypes.

### Theoretical prediction of imputation accuracy

Based on pedigree information, animal-specific imputation accuracy can be predicted for ungenotyped individuals. Predicted animal-specific imputation accuracies were derived using selection index theory [26], assuming genotype dosage is a trait with a heritability of 1 (assuming no genotyping errors), which provides the accuracy of a linear prediction of gene content. The accuracy (r) is derived as:

$$r=\sqrt{\left(P^{‒1}\times G\right)'\times G,}$$

where **P** is a square matrix with covariances (i.e., additive relationships) between the information sources, which are the genotyped relatives of the individual that is imputed; **G** is a vector with the covariances (i.e., additive relationships) between the information sources and the individual that is imputed.

## Results

Initial results showed that the animal-specific imputation accuracy was lower for AlphaImpute phased than for segregation analysis. Therefore, we report the results of segregation analysis for all scenarios. Differences between AlphaImpute phased and segregation analysis were assessed for the offspring scenarios only and are reported below.

### Animal-specific imputation accuracy

Table 2 shows that imputation of ungenotyped individuals based on family relationships was possible. Depending on the available family information, animal-specific imputation accuracies ranged from 0.42 to 0.72, and was 0.47 across all animals for scenario Real. Using the more commonly quoted statistic r_uncorrected_, for which mean gene content is ignored, the imputation accuracy across all animals was 0.80. The average imputation accuracy for individuals that had only their dam genotyped as most recent ancestor was 0.70, which was higher than expected because some had an offspring genotyped (9) and most had paternal half sibs genotyped.

**Table 2:** Average imputation accuracy (r) from segregation analysis of 1344 individuals for scenarios Real and SireMGS for different categories of individuals

	**Real**			**SireMGS**		
**Category**	**n**	**r**	**sd**^**2**^	**n**	**r**	**sd**
Both parents	14	0.72	0.08	43	0.73	0.07
SireMGS	62	0.61	0.13	1,258	0.60	0.12
DamPGS	6	0.63	0.08	0	-	-
Sire	241	0.59	0.13	24	0.54	0.14
Dam	23	0.70	0.09	0	-	-
Other	998	0.42	0.22	19	0.35	0.28
Total	1,344	0.47	0.22	1,344	0.60	0.13
r_uncorrected_^1^	1,344	0.80	0.06	1,344	0.84	0.04

Average imputation accuracies were split into categories of the closest recent ancestor genotyped and calculated over 10 replications; ^1^mean of imputation accuracy calculated as the correlation between true genotypes and imputed genotype dosages, both uncorrected for the mean gene content; ^2^standard deviation.

Including genotypes of sires and maternal grandsires in the reference population improved the animal-specific imputation accuracy across all animals from 0.47 (±0.22) to 0.60 (±0.13; r_uncorrected_ = 0.84) (Table 2). This substantial increase was obtained because, in the Real situation, few individuals had their sire and maternal grandsire genotyped, hence the large number of individuals that moved from category ‘Other’ to category ‘SireMGS’ when genotypes of sires and maternal grandsires were used (Table 2). In scenario SireMGS, the animal-specific imputation accuracy for the categories ‘Sire’ and ‘Other’ decreased compared to scenario Real. This is due to the considerable drop in the number of animals in these categories; the animals that remained in these categories had fewer relationships in the pedigree, which made it more difficult to impute them.

Table 3 shows that the addition of genotyped offspring to the reference population increased the animal-specific imputation accuracy considerably. If only one offspring was genotyped, the average animal-specific imputation accuracy across all animals increased from 0.57 (±0.12) to 0.73 (±0.07), if a second genotyped offspring was added, it increased further to 0.82 (±0.07), and if four genotyped offspring were added, it reached 0.92 (±0.05). Within each category of the most recent genotyped ancestor, the animal-specific imputation accuracy increased as the number of offspring genotyped increased. In particular, for the category ‘Other’, this increase was substantial: from 0.13 to 0.61, 0.77 and 0.91 if zero, one, two or four genotyped offspring were added, respectively. As the number of genotyped offspring increased, the animal-specific imputation accuracies of the different categories of the most recent genotyped ancestor became more similar, which indicates that with multiple genotyped offspring, information on the ancestors was less relevant.

**Table 3:** Average imputation accuracy (r) of 805 individuals with varying offspring information for different categories of individuals

		**Off0**		**Off1**		**Off2**		**Off4**	
**Category**	**n**	**r**_**segregation**_	**r**_**phased**_	**r**_**segregation**_	**r**_**phased**_	**r**_**segregation**_	**r**_**phased**_	**r**_**segregation**_	**r**_**phased**_
Both parents	25	0.70	0.66	0.78	0.78	0.84	0.83	0.93	0.94
SireMGS	756	0.57	0.54	0.73	0.68	0.82	0.77	0.92	0.88
DamPGS	0	-	-	-	-	-	-	-	-
Sire	15	0.50	0.48	0.69	0.68	0.80	0.78	0.93	0.87
Dam	0	-	-	-	-	-	-	-	-
Other	9	0.13	0.13	0.61	0.61	0.77	0.77	0.91	0.91
Total^1^	805	0.57	0.54	0.73	0.68	0.82	0.77	0.92	0.88
r_uncorrected_^2^	805	0.83	0.81	0.88	0.86	0.92	0.89	0.96	0.95

Results from both AlphaImpute with phasing (phased) and only segregation analysis (segregation) are reported; average imputation accuracies were split into categories of the closest recent ancestor genotyped and calculated over 10 replications; ^1^for AlphaImpute phased, the standard deviations for Total were 0.16, 0.13, 0.13, and 0.10 for scenarios Off0, Off1, Off2, and Off4, respectively; for segregation analyses, the standard deviations for Total were 0.12, 0.07, 0.07, and 0.05 for scenarios Off0, Off1, Off2, and Off4, respectively; ^2^mean of imputation accuracy calculated as the correlation between true genotypes and imputed genotype dosages, both uncorrected for the mean gene content.

### Percentage of (in) correct and not imputed SNP genotypes

Table 4 shows that the percentage of correctly imputed SNPs across all animals increased when the genotypes of sires and maternal grandsires were included, while the percentage of incorrectly imputed SNPs and of SNPs not imputed across all animals decreased. More SNPs were imputed when offspring were genotyped, which increased the percentage of correctly imputed SNPs, but if only one offspring was genotyped, the percentage of incorrectly imputed SNPs also increased. Having two or more genotyped offspring reduced the percentage of incorrectly imputed SNPs again.

**Table 4:** Average percentage of correct, incorrect or not imputed genotypes per individual for each scenario and for different categories of individuals

**Scenario**	**Imputed**	**Both parents**	**SireMGS**	**DamPGS**	**Sire**	**Dam**	**Other**	**Total**
*Segregation*								
Real	Correct	52.9	20.3	33.6	16.8	45.9	11.9	14.3
	Incorrect	0.0	0.2	0.4	0.2	0.7	0.2	0.2
	Not imputed	47.0	79.4	66.0	82.9	53.4	87.8	85.5
SireMGS	Correct	52.8	17.9	-	9.0	-	7.2	18.7
	Incorrect	0.0	0.1	-	0.1	-	0.2	0.1
	Not imputed	47.1	81.9	-	90.8	-	92.6	81.2
Off0	Correct	51.1	14.4	-	6.6	-	6.5	15.3
	Incorrect	0.0	0.1	-	0.1	-	0.2	0.1
	Not imputed	48.8	85.4	-	93.2	-	93.2	84.6
Off1	Correct	57.9	30.3	-	19.4	-	7.2	30.8
	Incorrect	0.2	1.15	-	1.3	-	0.1	1.1
	Not imputed	41.7	68.4	-	79.2	-	92.6	68.1
Off2	Correct	61.6	39.3	-	31.0	-	11.9	39.6
	Incorrect	0.2	0.9	-	1.4	-	0.2	0.8
	Not imputed	38.1	59.7	-	67.5	-	87.9	59.6
Off4	Correct	67.5	50.7	-	46.3	-	35.7	51.0
	Incorrect	0.1	0.3	-	0.2	-	0.2	0.3
	Not imputed	32.4	48.9	-	53.4	-	64.0	48.7
*Phased*								
Off0	Correct	40.8	12.4	-	4.9	-	4.7	13.1
	Incorrect	15.3	4.3	-	1.6	-	1.7	4.6
	Not imputed	43.8	83.2	-	93.3	-	93.5	82.3
Off1	Correct	71.8	43.4	-	22.1	-	7.0	43.5
	Incorrect	4.0	6.2	-	3.3	-	0.1	6.0
	Not imputed	24.1	50.3	-	74.6	-	92.8	50.5
Off2	Correct	73.4	60.1	-	37.1	-	11.8	59.6
	Incorrect	1.5	5.2	-	4.2	-	0.2	5.0
	Not imputed	25.0	34.6	-	58.7	-	87.9	35.4
Off4	Correct	87.4	78.4	-	61.7	-	35.9	78.0
	Incorrect	0.5	2.7	-	4.1	-	0.2	2.6
	Not imputed	12.1	18.8	-	34.2	-	63.8	19.4

For the offspring scenarios, results from both AlphaImpute with phasing (phased) and only segregation analysis (segregation) are given; average percentages were split into categories of the closest recent ancestor genotyped and calculated over 10 replications.

It should be noted that a relatively large number of genotypes were considered as not imputed because the amount of information in the data was not sufficient to impute one or both gametes (e.g., because of uncertainty around recombination events or uncertainty on which haplotype was inherited from one or both parents). However, these genotypes obtained genotype probabilities that were included to calculate the animal-specific imputation accuracy. The relatively high animal-specific imputation accuracies indicated that the genotype probabilities were reasonably good, although the SNPs were not imputed due to uncertainty.

### Segregation analysis outperformed AlphaImpute phased

Individuals with both parents genotyped and no offspring had an animal-specific imputation accuracy of 0.66 with AlphaImpute phased and of 0.70 with segregation analysis (Table 3), which is close to the expected accuracy of a parent average prediction $\left(\sqrt{0.5}=0.71\right)$. Table 3 shows that when both parents were genotyped but no offspring, AlphaImpute phased performed considerably poorer than segregation analysis, but when both parents and offspring were genotyped, both methods performed almost equally well. Segregation analysis performed better than AlphaImpute phased when only the sire or the sire and maternal grandsire were genotyped, regardless of the number of genotyped offspring (Table 3). When no ancestors were genotyped, both methods performed equally well based on animal-specific imputation accuracies.

With genotyped offspring, AlphaImpute phased imputed more SNPs correctly per individual than segregation analysis: across all animals, the percentage of correctly imputed SNPs was 13%, 20%, and 27% higher with AlphaImpute phased than with segregation analysis for scenarios Off1, Off2, and Off4, respectively (Table 4). However, AlphaImpute phased also imputed more SNPs per individual incorrectly: across all animals, the percentage of incorrectly imputed SNPs was 4.9%, 4.2%, and 2.3% higher with AlphaImpute phased than with segregation analysis for scenarios Off1, Off2, and Off4, respectively (Table 4).

### Theoretically predicted imputation accuracy

Predicted animal-specific imputation accuracies were derived using selection index theory for situations for which both parents were genotyped, one parent and one grandparent were genotyped, and when one parent was genotyped, and each of these situations was combined with no, one, two or four genotyped offspring, as shown in Table 5. When offspring were genotyped and included in the reference population for imputation, observed animal-specific imputation accuracies (Table 3) were higher than accuracies predicted based on selection index theory (Table 5), while in the Off0 scenario, the animal-specific imputation accuracies were similar to their theoretical predictions.

**Table 5:** Theoretically predicted imputation accuracy based on selection index theory

	**Number of genotyped offspring**			
	**0**	**1**	**2**	**4**
Both parents	0.71	0.76	0.79	0.84
SireMGS/DamPGS	0.56	0.66	0.73	0.80
Sire/Dam	0.50	0.63	0.71	0.79

Predicted for situations with both parents genotyped, one parent and one grandparent genotyped (SireMGS/DamPGS), or one parent genotyped (Sire/Dam) and with different numbers of genotyped offspring.

### SNP-specific imputation accuracy

**Figure 1:**
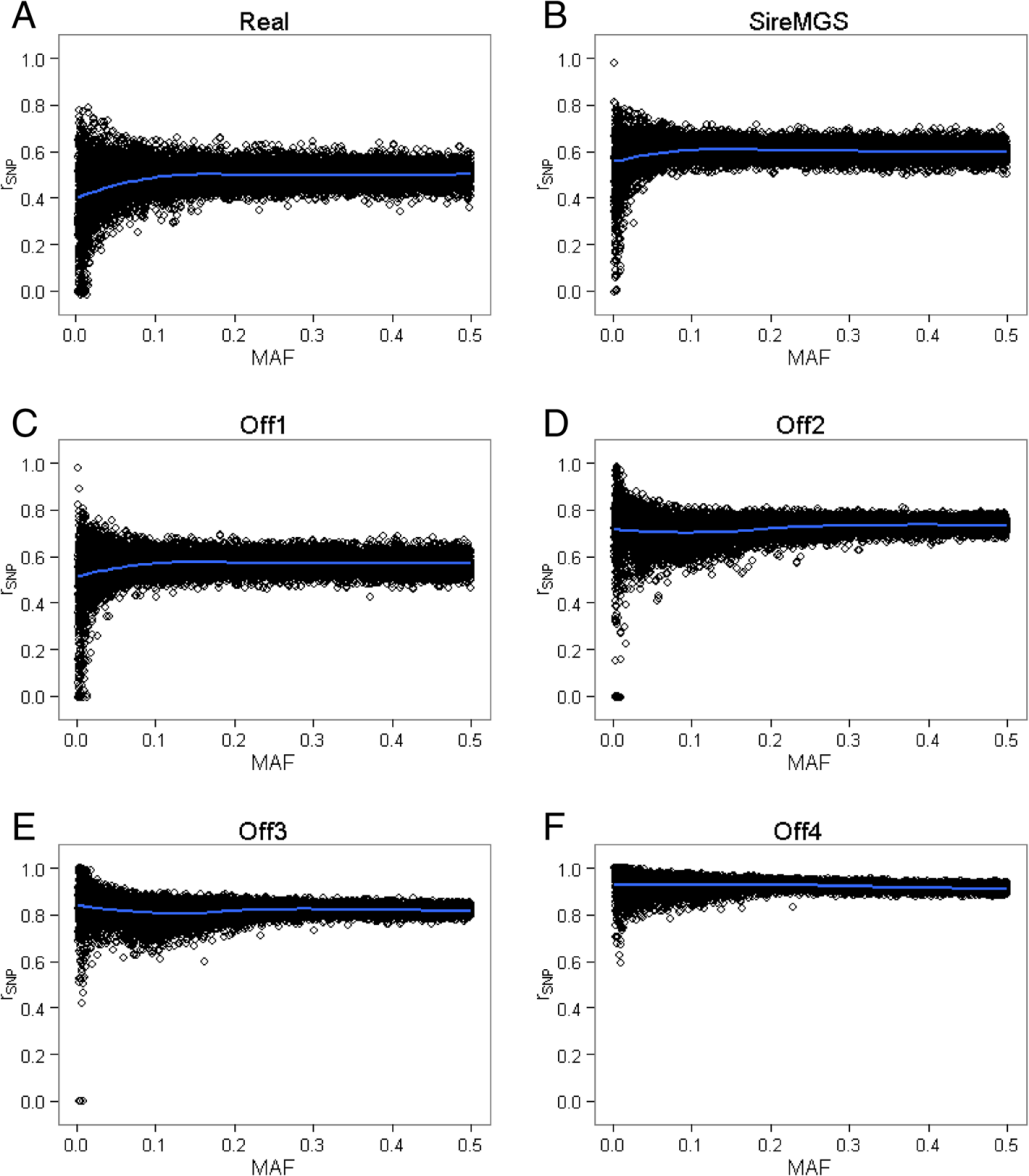
**Imputation accuracy by SNP (r**_**SNP**_**) plotted against the minor allele frequency (MAF) for each scenario.** Imputation accuracy was defined as the correlation of true genotypes with imputed genotype dosages by SNP and was calculated across 10 replicates (2000 SNPs × 10 replicates) for scenario Real **(A)**, SireMGS **(B)**, Off0 **(C)**, Off1 **(D)**, Off2 **(E)**, and Off4 **(F)**. The blue curves were obtained by fitting a nonparametric local regression (LOESS).

SNP-specific imputation accuracies increased as the number of close relatives genotyped increased, as expected (Figure 1). More interesting was the fact that SNP-specific imputation accuracy depended less on MAF when offspring were genotyped. As shown in Figure 1, the imputation accuracy of SNPs with low MAF increased considerably when offspring were genotyped. Figure 1 is different to figures reported in studies that impute genotypes from low-density to higher-density SNP panels, in which LD information from typed SNPs can be used for imputation. In such cases, quite a few imputed SNPs are in complete LD with typed SNPs, and thus have a SNP-specific imputation accuracy of 1 regardless of the MAF. In this study, there were no typed SNPs and LD information was not used, thus only a few SNPs had an imputation accuracy equal to 1.

**Figure 2:**
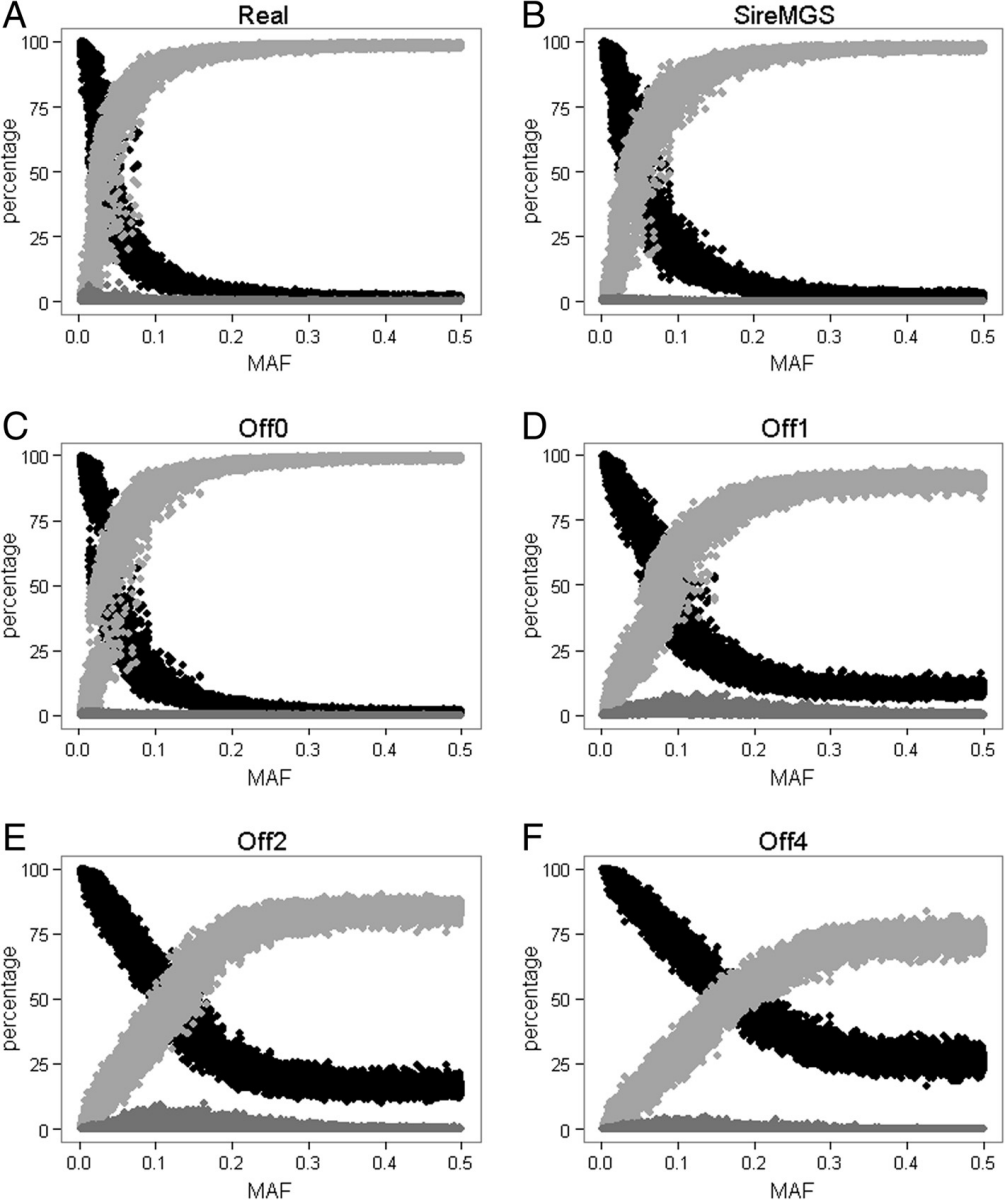
**Percentage of (in)correct and not imputed genotypes by SNP, plotted against the minor allele frequency (MAF) for each scenario.** Percentages of correctly imputed genotypes (in black), incorrectly imputed genotypes (in dark grey), and not imputed (in light grey) genotypes by SNP, plotted against MAF for scenario Real **(A)**, SireMGS **(B)**, Off0 **(C)**, Off1 **(D)**, Off2 **(E)**, and Off4 **(F)** and calculated across 10 replicates (2000 SNPs × 10 replicates).

As in other studies, the percentage of correctly imputed SNPs and of SNPs not imputed depended strongly on the MAF (Figure 2). Results showed that a higher MAF made it difficult to impute SNPs with high certainty and therefore many of those genotypes were not imputed. If there were no genotyped offspring, the percentage of SNPs not imputed plateaued at 95-100% for SNPs with a MAF greater than 0.2 (Figure 2A, 2B, and 2C). With four genotyped offspring, this percentage plateaued at 60-80% for SNPs with a MAF greater than 0.3 (Figure 2F). Because more genotypes were imputed when offspring were genotyped, there was also more chance that they were imputed incorrectly and, therefore, the percentage of incorrectly imputed SNPs was higher with genotyped offspring (Figure 2C versus Figure 2D). However, this percentage decreased again when more offspring were genotyped (Figure 2C versus Figure 2F).

## Discussion

The objective of this study was to investigate the accuracy of imputation of non-genotyped individuals. The results showed that sufficient accuracies can be obtained when multiple offspring are genotyped. This enables re-use of valuable phenotypes from historical datasets for, e.g. GWAS or genomic prediction. Usually, such datasets with valuable phenotypes are small and in such cases, adding phenotyped individuals with imputed genotypes can have a relatively larger impact on the power of GWAS or on the improvement of the accuracies of genomic prediction.

### Imputation method

AlphaImpute was designed as a flexible method to impute genotyped and ungenotyped individuals, performs well for very low-density scenarios [15], and provides informative output, such as genotype dosage based on genotype probabilities. VanRaden et al. [10] showed that imputation with FindHap and FImpute performed well for imputation of ungenotyped dams with four or more genotyped offspring. However, in our study, with four or less genotyped offspring, FindHap resulted in lower imputation accuracy than AlphaImpute (ranging from 0.13 to 0.38 based on one replicate of each scenario; results not shown). Also Pimentel et al. [5] reported that FindHap resulted in lower imputation accuracies than AlphaImpute for imputation of ungenotyped individuals with one genotyped offspring. FImpute did not accept ungenotyped individuals with less than four genotyped offspring, and both FindHap and FImpute do not provide genotype probabilities as output. The genotype probabilities provide an implicit measure of uncertainty in the imputation that can be taken into account in further analyses based on imputed genotypes, such as GWAS or genomic prediction. It has been shown that using genotype dosage instead of the most likely genotype for selection candidates increased the reliability of genomic predictions [25,27]. For GWAS, it has also been suggested to include the uncertainty of imputed genotypes to improve power [28-31]. Therefore, output in the form of probabilities should become a standard output of imputation programs in the future, to enable the inclusion of imputation uncertainty into further analyses.

In the segregation analysis approach, AlphaImpute was used without phasing, which resulted in imputation using only simple imputation rules and segregation analysis, and thus did not use long range phasing and haplotype library imputation [32]. The phasing results from AlphaImpute with phasing showed only minor errors compared to the true phase of the genotypes (results not shown) and, thus, hardly affected the imputation results. AlphaImpute with phasing, which used the haplotype library, resulted in more correctly imputed genotypes per individual but also in more incorrectly imputed genotypes per individual, which led to a lower animal-specific imputation accuracy than segregation analysis (Table 3). The increase in incorrectly imputed SNPs per individual is due to the long-range haplotype imputation algorithm in AlphaImpute, which allows a certain mismatch (set to 1% in this study) between SNPs of the most likely haplotype from the haplotype library and (in a previous step imputed) genotypes of the imputed individual. It is likely that this has a smaller effect in populations with larger effective population sizes, because in such populations, larger haplotypes have a lower probability of incorrectly fitting by chance. Pimentel et al. [5] showed that in scenarios with high LD, such as in dairy cattle, imputation with simple segregation rules performed better than other imputation programs they tested (FindHap, AlphaImpute, and a two-step approach using simple imputation rules followed by fastPHASE). They also showed that imputation using LD information was as successful as segregation analysis when one chromosome had at least 300 unambiguously imputed SNPs of the 2000 SNPs it contained. This indicates that the main issue with imputation of ungenotyped individuals is the limited number of SNPs that can be imputed unambiguously and these might not be evenly distributed across the genome. Therefore, imputation algorithms that are specifically designed for imputation of ungenotyped individuals are needed. If such algorithms make optimal use of all the features of genotype data, such as phase and LD information, without introducing errors, they should lead to higher imputation accuracies than segregation analysis.

### Animal-specific imputation accuracy

In the literature, several definitions of imputation accuracy are used. As pointed out by Hickey et al. [33] and empirically shown by [11,34], the widely used percentage of correctly imputed SNPs depends on the MAF, and the correlation between the true genotype and the imputed genotype (or dosage) is a better measure of the quality of imputation. However, for the animal-specific imputation accuracy, different SNPs have different MAF, and thus also a distribution with a different mean, while a Pearson correlation assumes that the correlated variables are bivariate normally distributed. Therefore, we corrected genotypes for the mean gene content of each SNP, as suggested by Mulder et al. [25]. The uncorrected correlation (r_uncorrected_) was overestimated as a result of bias due to differences in MAF across loci. The difference between the two statistics was particularly large when the imputation accuracy was low. When the imputation accuracy approached 1, the difference between the two statistics reduced towards 0.

For individuals with both parents genotyped, imputing their genotypes as the average of its parents resulted in animal-specific imputation accuracies close to the expected accuracy for a parent average $\left(\sqrt{0.5}=0.71\right)$ when we corrected for mean gene content (0.70). Without this correction, the imputation accuracy was much higher (0.87) due to the aforementioned bias. This indicates that correction for mean gene content makes it possible to compare observed imputation accuracies with those calculated based on selection index theory. Therefore, we conclude that the animal-specific imputation accuracy should indeed be computed after correction of genotypes for mean gene content, as suggested by Mulder et al. [25].

### Use of imputed genotypes in further analyses

An important question is whether the use of phenotypes from imputed animals is advantageous, for example, in GWAS or genomic prediction. This question is not specifically addressed in the simulations presented here, but has received some attention in the literature. For example in human GWAS studies, inclusion of predicted genotypes for phenotyped individuals can increase the power of GWAS when close relatives are genotyped [1-3]. Chen et al. [1] did not observe a clear relationship between imputation accuracy and improvement in power, but there appeared to be a trade-off between imputation accuracy and sample size. Even when imputation was relatively inaccurate, adding phenotyped individuals with predicted genotypes increased power compared to not using these individuals in the study [2], which suggested that imputation of ungenotyped individuals with phenotypes is worthwhile for GWAS.

For genomic prediction, a few studies have shown that the accuracy of genomic predictions can be improved when phenotyped individuals with imputed genotypes are added to the reference population of genotyped individuals [4,5]. Pimentel et al. [5] showed that improvement in the accuracy of genomic prediction was in general larger when heritability of the trait was lower, and when the initial reference population was smaller. Pszczola et al. [6] showed no significant improvement in accuracy of genomic prediction from adding imputed genotypes due to low imputation accuracy, although a slight improvement was seen with low heritabilities. The authors suggested that the low imputation accuracy was caused by the population structure, as only sires and maternal grandsires had genotypes. Likewise, Hickey et al. [4] indicated that imputation of ungenotyped individuals that are distantly related to the genotyped population do not contribute much to the improvement in accuracy of genomic prediction. Thus, for genomic prediction, the addition of animals with phenotypes and imputed genotypes to the datasets increases accuracy also, although the magnitude of this improvement depends on heritability of the trait, imputation accuracy, and size and structure of the population [4-6].

Another important question is whether an imputation step is actually necessary to improve the accuracy of genomic predictions. The so-called one-step approach conveniently combines genotyped and ungenotyped animals through a relationship matrix, called the H-matrix [35,36]. Using this one-step approach, Christensen and Lund [35] and Hickey et al. [4] showed that including many additional phenotyped animals without genotypes into the reference population improved the accuracy of genomic predictions compared to using only the genotyped individuals as the reference population. However, it should be kept in mind that the one-step approach applies an implicit linear imputation method, similar to imputation using mixed model equations suggested by Gengler [37]. In other words, the implicit imputation in the one-step approach may be less accurate than imputation using more sophisticated methods and may lead to a loss in accuracy in genomic prediction.

Hickey et al. [4] compared two strategies to include phenotypes of ungenotyped individuals in the reference population, i.e., prediction using an explicit imputation step and prediction using an H-matrix. They found very small differences in accuracy of genomic predictions between the two methods. Unfortunately, neither the imputation accuracy of the ungenotyped individuals, nor the relationships between genotyped and ungenotyped individuals were provided in Hickey et al. [4], and linear imputation might have been sufficient with their data. In the current study, we have shown that imputation accuracies from segregation analysis are higher than those predicted by selection index theory when offspring are genotyped. Therefore, we also expect a higher accuracy of genomic predictions, when the genotypes of such animals are imputed explicitly using sophisticated methods.

## Conclusions

Ungenotyped individuals from a historical dairy cattle population could be imputed with an imputation accuracy, i.e. correlation of true genotype with the imputed genotype dosage corrected for mean gene content, of 0.60 when genotypes of sires and maternal grandsires were available. When the more common correlation between genotype dosage and true genotype (r_uncorrected_) was used, an imputation accuracy of 0.84 was obtained. With genotyped offspring, imputation accuracies increased towards 0.92 (r_uncorrected_ = 0.96) with four offspring and MAF and ancestor genotypes became less relevant for imputation. Basic segregation rules appeared to be the best currently available imputation method for ungenotyped individuals. Therefore, imputation algorithms specifically designed for ungenotyped individuals using LD and family information need to be developed in order to further increase imputation accuracies by using all features of genotype data. Comparison of our empirical animal-specific imputation accuracies with predictions based on the selection index theory suggested that not correcting for mean gene content considerably overestimates the true animal-specific imputation accuracy. In conclusion, imputation of ungenotyped individuals can help to include valuable phenotypes in GWAS or genomic predictions, in particular when genotyped offspring are available.

## Competing interests

The authors declare that they have no competing interests.

## Authors’ contributions

ACB participated in the design of the study, carried out the analysis, was involved in discussions, prepared and drafted the manuscript. JMH participated in the design of the study, developed and adapted the software used for imputation, was involved in discussions, and helped to draft the manuscript. MPLC participated in the design of the study, was involved in discussions and helped to draft the manuscript. RFV participated in the design and coordination of the study, was involved in discussions, and helped to draft the manuscript. All authors read and approved the final manuscript.
